# Virtual reality as a means to explore assistive technologies for the visually impaired

**DOI:** 10.1371/journal.pdig.0000275

**Published:** 2023-06-20

**Authors:** Fabiana Sofia Ricci, Alain Boldini, Xinda Ma, Mahya Beheshti, Duane R. Geruschat, William H. Seiple, John-Ross Rizzo, Maurizio Porfiri

**Affiliations:** 1 Department of Biomedical Engineering, New York University Tandon School of Engineering, Brooklyn, NY, United States of America; 2 Center for Urban Science and Progress, New York University Tandon School of Engineering, Brooklyn, NY, United States of America; 3 Department of Mechanical and Aerospace Engineering, New York University Tandon School of Engineering, Brooklyn, NY, United States of America; 4 Department of Rehabilitation Medicine, New York University Langone Health, New York, NY, United States of America; 5 Wilmer Eye Institute, The Johns Hopkins University School of Medicine, Baltimore, Maryland, United States of America; 6 Lighthouse Guild, New York, NY, United States of America; 7 Department of Ophthalmology, New York University Grossman School of Medicine, New York, NY, United States of America; 8 Department of Neurology, New York University Langone Health, New York, NY, United States of America; Massachusetts Institute of Technology, UNITED STATES

## Abstract

Visual impairment represents a significant health and economic burden affecting 596 million globally. The incidence of visual impairment is expected to double by 2050 as our population ages. Independent navigation is challenging for persons with visual impairment, as they often rely on non-visual sensory signals to find the optimal route. In this context, electronic travel aids are promising solutions that can be used for obstacle detection and/or route guidance. However, electronic travel aids have limitations such as low uptake and limited training that restrict their widespread use. Here, we present a virtual reality platform for testing, refining, and training with electronic travel aids. We demonstrate the viability on an electronic travel aid developed in-house, consist of a wearable haptic feedback device. We designed an experiment in which participants donned the electronic travel aid and performed a virtual task while experiencing a simulation of three different visual impairments: age-related macular degeneration, diabetic retinopathy, and glaucoma. Our experiments indicate that our electronic travel aid significantly improves the completion time for all the three visual impairments and reduces the number of collisions for diabetic retinopathy and glaucoma. Overall, the combination of virtual reality and electronic travel aid may have a beneficial role on mobility rehabilitation of persons with visual impairment, by allowing early-phase testing of electronic travel aid prototypes in safe, realistic, and controllable settings.

## Author summary

Testing electronic travel aids under development is an outstanding research area, due to the rapid growth in the number of people with visual impairment. For decades, different technologies have been employed to improve the mobility of persons with visual impairment, but suitable and easy-to-use solutions have not yet been established. In this study, we propose the use of virtual reality for early-phase testing of electronic travel aids in safe, realistic, and controllable settings. We demonstrate the approach using an haptic feedback device developed in-house in the form of a belt. Our device can be simply wear by a user, providing free hands and real-time operation. The approach offers the combined possibility of designing highly realistic, urban environments and of simulating different forms of visual impairment on healthy subjects. Our integrated wearable electronic travel aid/virtual reality system establishes a novel assistive framework to mitigate the consequences of visual impairment. We envision this framework could improve training, reduce rehabilitation, and abate societal costs, while creating an engaging and compelling experience for persons with visual impairment.

## Introduction

Visual impairments (VIs) are a form of disability that have an increasing and alarming prevalence on a global scale. As of 2020, 596 million people globally experiencing moderate or severe VI. This number is expected to double by 2050, as a consequence of the increase in life expectancy [1, 2]. As we move through the 2020 decade, the ‘baby-boom’ generation will turn over 65 years old; it is predicted that this demographic change will cause a rapid increase in the number of people affected by VIs [1].

VIs often cause severe consequences for affected persons. Not only do these disabilities often yield poor quality of life, mostly associated with the reduction in mobility, but also bear other important health consequences. Persons with VI may experience increased odds of falls, hip fractures, obesity, depression, and even death [3–5]. Further, visual impairments bring about a variety of social issues, including unemployment and social isolation [3, 6, 7]. Consequently, the direct and indirect costs of VIs, due to medical expenses and reduction in productivity, respectively, are among the largest for all disabilities [8].

A variety of solutions have been proposed to mitigate the reduction in mobility due to VI. However, the two systems that are currently most adopted by persons with VI, white canes and guide dogs, present a series of limitations that hinder their use. White canes require a considerable amount of training and cannot identify obstacles that are higher than the hips. On the other hand, guide dogs are expensive to maintain and train. Both of these solutions may cause social anxiety in individuals who adopt them, as they are easily noticeable. As a result, only 2% of persons with VI actually utilizes any of these solutions [9].

With technological advances, new navigation aids have become available to persons with VI, in the form of electronic travel aids (ETAs). These devices integrate sensing and feedback systems of various natures. Sensing ranges from computer vision technology to scanning devices and microphones [10–13], while feedback systems span from audio devices to haptic accessories [14–17]. These sensory substitution systems rely on data acquired from the sensing system to infer the presence of obstacles in the surroundings of the user, who can negotiate obstacles based on the information relayed by the feedback system.

Despite the increasing number of ETAs for improving mobility of persons with VI, their adoption remains low [18]. The most challenging step in the technological development of ETAs is surpassing the prototype phase, by testing the device on a sufficiently large cohort of human subjects in realistic conditions to refine designs. Difficulties in recruiting subjects, differences in pathophysiology of different VIs, and varying degrees of severity of VIs are all factors that exacerbate the process of human testing. For example, [18] reported that just 42% of the most popular technological canes and 50% of the robots for mobility were tested with persons with VI. In many cases, only blindfolded sighted individuals participated in the testing of the technological canes, and the testing of robots was performed with a very small sample sizes (two-three subjects).

Until recently, the lack of specific certification procedures for ETAs has been a major hurdle, not allowing for standardized routes for clearance and approval toward commercialization [19–21]. ETAs are often complex and even counter-intuitive, such that their use requires a significant amount of training [18]. To address these problems, there is an immediate need for a platform that can: i) support the development, testing, and refinement out of the prototype stage of ETAs, targeted to end-users; and ii) train persons with VIs to use ETAs within realistic scenarios, while limiting their risk of injury in the process [22–24].

Here, we propose that virtual reality (VR) can provide a novel testing and training platform for ETAs. From the testing point of view, VR offers a practical way to assess whether an ETA feedback system under development can promptly convey environmental information to users in order for them to engage in safe and efficient travel [25]. Additionally, VR allows for an accurate simulation of symptoms associated to different forms of VI with a high level of realism. This advantage is particularly useful as it allows for engagement of fully sighted subjects with simulated VI during the first testing phase of an ETA, thereby facilitating the recruiting process. From the training point of view, VR enables the creation of virtual environments that resemble real-life scenarios and are free of many of the risks associated with navigation in the real world. Practicing in a high realistic and safe virtual environment can enhance engagement and reduce the stress experienced by novice trainees, thus shortening the overall training period. The possibility of exacerbating an underlying pathology can also improve training of persons affected by mild/moderate VI and prepare them for lost functions in progressive conditions.

To the best of our knowledge, smart white canes devices have been connected to VR environments as controllers for persons with VIs [26–28], but no study has considered the use of VR tasks for the systematic testing of ETAs. There are also VR platforms for orientation and mobility (O&M) training where trainees, with and without simulated VI, can walk through realistic scenarios safely while listening to sounds such as vehicles, stores, ambient noise, etc. [25, 29–31]. Not only did these studies confirm the accuracy of the simulation of VIs in VR, but also they demonstrated the efficacy of virtual training, whereby acquired mobility skills generalized to real environments. None of these efforts included the training of O&M skills with an ETA.

In this manuscript, we demonstrate the potential of VR to assess the performance of an ETA in improving the mobility of persons with VIs, a fundamental step in both ETA testing and training. Specifically, we conduct an experiment with 48 healthy subjects performing an obstacle avoidance task in VR while experiencing different simulated VIs. Subjects perform the task in two conditions: with and without an ETA, consisting of a wearable haptic feedback device developed by our group [32–34]. By comparing the performance of subjects with or without the ETA, we quantify the benefit that the device provides to mobility. Such information can be utilized for refining the design of ETAs toward commercialization, as well as for the evaluation of trainees’ progress in the use of an ETA.

## Results

### Interfacing VR and ETAs

We devised an experimental platform to demonstrate that a well-conceived VR environment can provide the ideal framework for development, initial evaluation, refinement, and training with ETAs. Indeed, VR offers the possibility to design safe, controllable, and repeatable trials where accurate simulations of different forms of VIs can be implemented and a variety of conditions and situations can be investigated. We interfaced the ETA with our VR system and designed an obstacle avoidance task in virtual environment, toward assessing the ability of our ETA to improve user mobility in VR.

**Fig 1:**
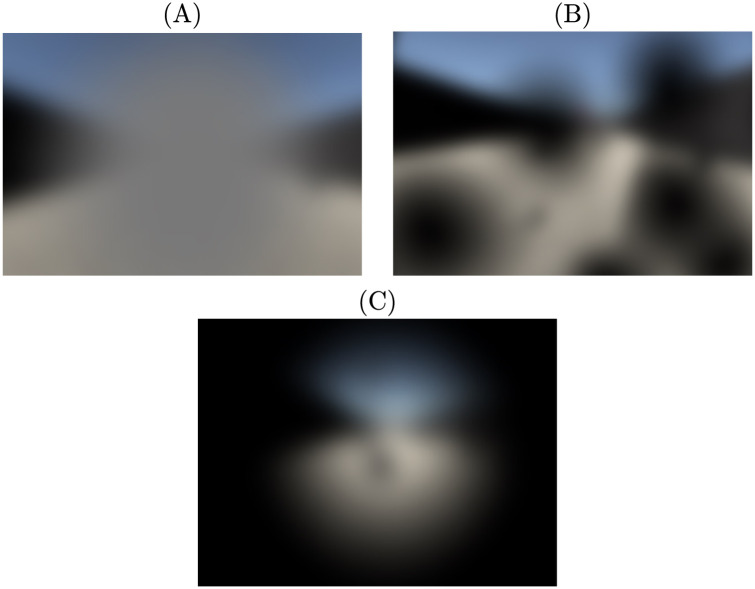
Visual impairment simulation in virtual reality: (A) age-related macular degeneration (AMD), (B) diabetic retinopathy (DR), and (C) glaucoma.

The VR platform could simulate three VIs, which are the leading causes of blindness and low vision in the United States: age-related macular degeneration (AMD), diabetic retinopathy (DR), and glaucoma, see Fig 1 [35, 36]. AMD is an eye disorder associated with aging that affects the macula, the central part of the retina that allows the eye to see fine details [37]. Peripheral vision, however, is unaffected by this pathology. AMD results in the damage of sharp and central vision, which is needed for seeing objects clearly and performing common daily tasks [37]. Diabetic retinopathy (DR) is a common complication of diabetes and is the leading cause of blindness in American adults [38]. DR is characterized by progressive damage to the blood vessels of the retina, which in advanced stages of the disease can cause dark, floating spots or streaks that look like cobwebs in the field of vision [38]. Glaucoma is a group of eye diseases that occurs when the normal fluid pressure inside the eyes slowly rises [39], leading to damage of the optic nerve. Glaucoma symptoms consist of loss of peripheral vision and/or blind spots [39].

Simulation of the three VIs was achieved through the combination of two tools in Unity, see Methods “Virtual reality software”. We considered simulations of late-stage, severe VIs based on the extent of the visual field loss and on the intensity of symptoms such as blurred vision, reduced contrast, and glare light [50, 51]. The severe stage was chosen to provide an assessment of the benefits in the use of the ETA when the residual vision is extremely limited [40, 41].

**Fig 2:**
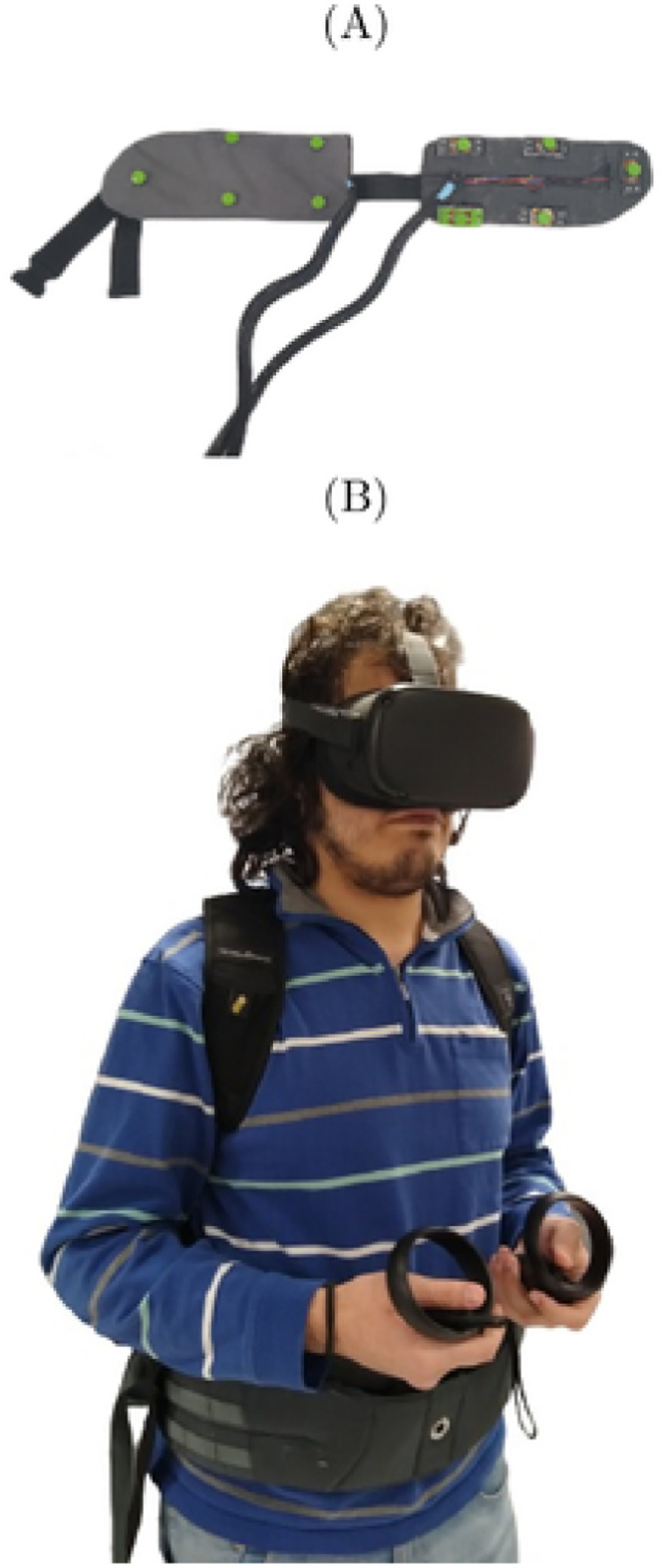
(A) Picture of the ETA tested in this study and (B) subject wearing the ETA together with the VR device.

The ETA tested in this study consisted of a haptic feedback wearable device, comprised of a belt with ten discrete piezoelectric actuators arranged as in Fig 2 to form a grid on the belt [32, 40]. The actuators on the belt can provide a vibrotactile stimulation on the users’ abdomen to alert them about the presence and location of obstacles in their immediate surroundings in the VR environment, see Methods “Obstacles detection in VR”.

In its original configuration, the ETA was connected to a camera and a processing unit that were combined to form a computer vision system able to detect obstacles. This computer vision system partitioned the recorded scene into a grid of rectangular capture fields, mirroring the arrangement of the actuators on the belt. If an obstacle was detected in one of these rectangles, the corresponding actuator on the belt started vibrating. The amplitude and frequency of the vibration were modulated based on the distance of the obstacles, whereby closer obstacles were signaled by stronger vibrations at higher frequencies.

When the ETA was interfaced with the VR platform, the function previously performed by the computer vision system was transferred to a Unity function called Raycast, which detects objects in VR that come in contact with rays emanated from the user camera (see Methods Obstacles detection in VR). Anytime an obstacle was detected by the Raycast function, the actuators to be activated on the belt were selected based on the relative position of the obstacle with respect to the user in VR.

### Experimental design and hypothesis testing

**Fig 3:**
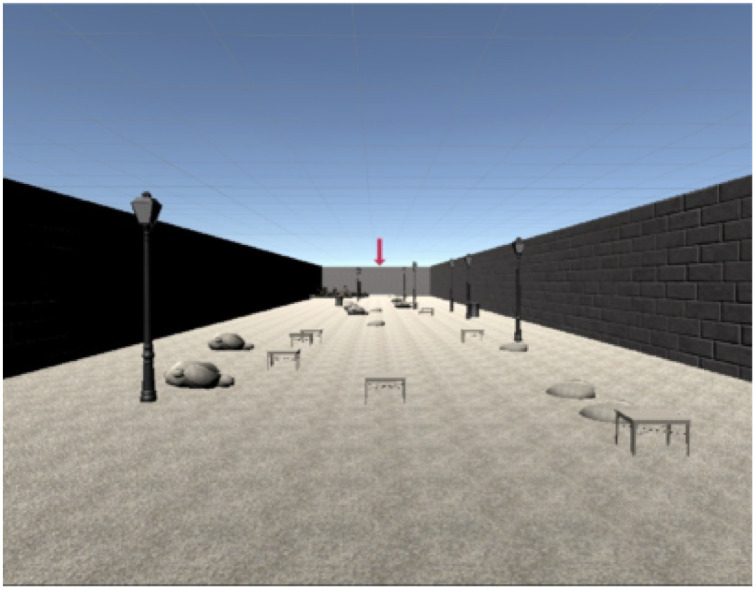
Example of the virtual reality environment used for the obstacle avoidance task.

We designed an experiment in which subjects perform an obstacle avoidance task in VR with or without the aid of the ETA. The task consisted of crossing a rectangular park in the least time possible, while trying to avoid hitting obstacles (benches, streetlamps, fences, and trash bins, see Fig 3). The ETA provided information about the presence of obstacles along the way through vibration feedback on the abdomen, helping prevent potential collisions. On the other hand, the Oculus Touch controller vibrated any time a collision occurred. This functionality reproduced the tactile sensation associated with collisions and provided feedback for those performing the task without the ETA, who would otherwise have no possibility of correcting their path.

We considered three different simulated VIs (AMD, DR, and glaucoma). Each subject performed the task with and without the aid of the ETA, for all the simulated VIs. The order of the condition tested and of the trials was counterbalanced among participants to prevent potential biases due to fatigue and training. Similarly, the position of obstacles in the environment was randomized to avoid learning effects. During the experiment, two metrics were acquired to measure subjects’ performance: time to complete the path and number of collisions, see Methods “Metric quantification in VR”.

We expected that the ETA would have a positive impact on both metrics, reducing the time to complete tasks and the number of mobility incidents. We anticipated that subjects would complete the navigation path faster when aided by the ETA, as wearing the belt should make them more confident. Similarly, the vibration feedback provided by the ETA should induce participates to avoid the obstacles more promptly, thus reducing the number of collisions. Specifically, through experiments, we tested the following hypotheses:

H1. The ETA can successfully assist subjects with simulated VI by reducing the time taken to complete the obstacle avoidance task. In other words, the completion time of a subject with a simulated VI is higher when they are not using the ETA.
H2. The ETA can successfully assist subjects with simulated VI by reducing the number of collisions during the obstacle avoidance task. In other words, the number of collisions of a subject with a simulated VI is higher when they are not using the ETA.

To test hypothesis H1, we performed a two-way ANOVA including random effects on the time taken by each participant to complete the task with and without the ETA for each VI. To test hypothesis H2, we performed a *χ*^2^ test on the total number of collisions of each participant while completing the tasks with the ETA compared to no ETA. To elucidate potential variations in the effectiveness of the belt on different VIs, we performed a Kruskal-Wallis test on the difference in the number of collisions of each participant while completing the tasks with and without the ETA as a function of the VI. When appropriate, post-hoc comparisons were conducted to detail pairwise differences. In S1 Text, we study the correlation between the completion time and the number of collisions, to understand whether subjects prioritized one metric over the other.

### Mobility performance assessment

**Fig 4:**
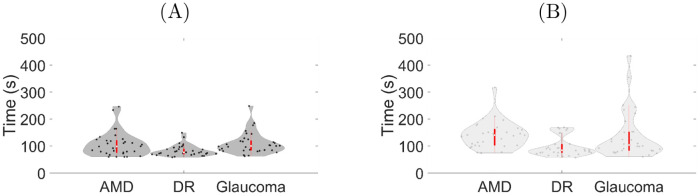
Time taken to complete the obstacles avoidance task: (A) with the belt and (B) without the belt while experiencing age-related macular degeneration (AMD), diabetic retinopathy (DR), and glaucoma. The area enclosed by the red rectangle inside each violin plot represents the corresponding box plot. The bold white dot inside each box details the median, and the bottom and top of the box identify the first and third quartiles, respectively. The area of a violin plot corresponds to the empirical probability density of the data.

**Fig 5:**
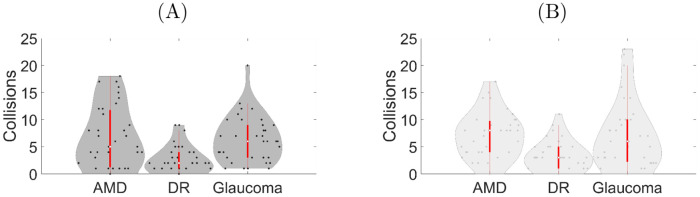
Number of collisions: (A) with the belt and (B) without the belt while experiencing age-related macular degeneration (AMD), diabetic retinopathy (DR), and glaucoma. The area enclosed by the red rectangle inside each violin plot represents the corresponding box plot. The bold white dot inside each box details the median, and the bottom and top of the box identify the first and third quartiles, respectively. The area of a violin plot corresponds to the empirical probability density of the data.

The experimental results of the ETA on the time taken to complete the obstacles avoidance task and on the number of collisions for the different simulated VI (AMD, DR, and glaucoma) are reported in Figs 4 and 5, respectively.

Quantitatively, the completion time was reduced due to the use of the ETA for the three VIs (Fig 4). We also note that the VIs simulations lead to quantitatively different performance, with DR posing the least strain on the completion time (median value around 75s) and AMD the strongest one (median value around 150 s without the belt and 100 s with the belt).

Although to a lesser extent than the time to completion, the number of collisions was quantitatively affected by use of the ETA. Specifically, for AMD and glaucoma the ETA produced larger median values than DR (Fig 5).

From the ANOVA performed on the time taken to complete the virtual obstacle avoidance task with and without the ETA, we verified hypothesis H1 that our ETA positively impacted participants’ speed (*F*_1,155_ = 17.9252, *p* < 0.001). We also found that the effect of the VI on the completion time was significant (*F*_1,155_ = 10.4161, *p* < 0.001), but not the combination of ETA and VI (*F*_2,155_ = 0.9944, *p* = 0.3723). From a post-hoc analysis on VIs, we found a significant difference on the completion time between AMD and DR and between DR and glaucoma (*t*_155_ = 4.047, *p* < 0.001 and *t*_155_ = −3.851, *p* < 0.001, respectively), but not between DR and glaucoma (*t*_155_ = 0.196, *p* = 0.979).

**Fig 6:**
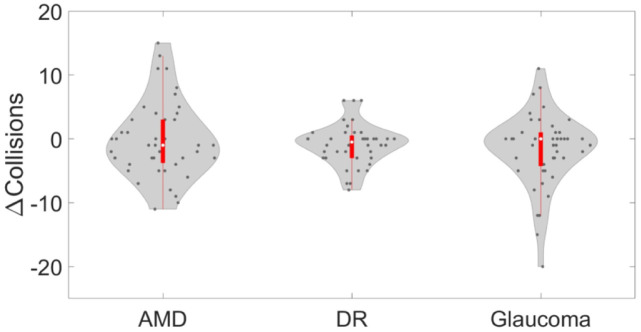
Effect of the ETA on the difference in the number of collisions occurred while experiencing experiencing the following VIs: Age-related macular degeneration (AMD), diabetic retinopathy (DR), and glaucoma. The area enclosed by the red rectangle inside each violin plot represents the corresponding box plot. The bold white dot inside each box details the median, and the bottom and top of the box identify the first and third quartiles, respectively. The colored area of a violin plot corresponds to the empirical probability density of the data. A negative value indicates a reduced number of collisions when the subjects used the ETA.

From the *χ*^2^ test performed between the two conditions, “with the ETA” and “without the ETA”, we verified hypothesis H2 that our ETA reduced the total number of collisions ($\chi_{361}^{2}=540.68$, *p* < 0.001). Considering the difference in the number of collisions with and without the ETA, we found no variation with respect to the VI (Kruskal-Wallis $\chi_{2}^{2}=0.437,p=0.803$), see Fig 6.

In S1 Text, we showed that the completion time is positively correlated with the number of collisions, suggesting the importance of individual skills on the performance.

## Discussion

Vision loss is a leading cause of disability among aging adults worldwide, primarily resulting from eye diseases such as age-related macular degeneration (AMD), diabetic retinopathy (DR), and glaucoma. As life expectancy continues to rise, the incidence of vision impairment (VIs) is also projected to grow, with global incidence of moderate-to-severe VIs doubling over the next 30 years.

Safe and independent navigation is often an extremely difficult task for persons affected by VI, due to the lack of sufficient environmental information for obstacle avoidance and orientation. To support orientation and mobility of persons affected by VI, a wide range of novel navigation systems has become available. These devices, known as electronic travel aids (ETAs), may provide critical information to the user, such as the distance, position, and relative speed of an obstacle, which is normally gathered by the visual system and is necessary for obstacle negotiation during navigation. Despite the promise of these ETAs, their use is still very limited. In fact, the adoption of ETAs is mainly affected by: i) the difficulties in passing the prototype phase, through testing the device with persons with VI, and ii) the need for appropriate training required to master their use [41, 42].

In this study, we proposed a new platform for testing, refining, and training with ETAs in virtual reality (VR). The platform allows to overcome many of the difficulties experienced in the first phases of ETA development by providing the ideal environment for testing the ETA in a controlled setting. In particular, VR offers the possibility of simulating a wide range of VIs at different levels of severity, thus solving the issue of recruiting a large number of persons with VI for testing from the very initial phases of the development. In fact, the number of persons with VI which could be expected to be involved in a series of research studies may be small, due to the possibility that a large fraction of them is unknown to services and/or do not wish to self-identify as person with VI [43]. We acknowledge that interfacing VR with different types of ETAs may be a challenging and time-consuming task. However, the possibility of interfacing an ETA with VR affords a reduction in time spent in recruiting, especially through the simulation of different VIs for testing on healthy subjects. Further, simulations in VR offer an incredible opportunity to test or train with an ETA in very different environmental conditions, such as with different lighting, to fully characterize the effect of the ETA and afford a safe training even in conditions that would be rare or particularly challenging in the real world.

We put forward a first example of our concept by integrating VR with an ETA previously developed by our group [32, 40]. Specifically, the ETA provided a vibrotactile stimulation on the abdomen of the user to inform them of the presence of obstacles in their surroundings in the virtual environment. The system was evaluated through a hypothesis-driven experiment where 48 healthy participants were asked to perform an obstacles avoidance task in VR. During the experiment, participants were asked to navigate in a rectangular park as fast as possible while trying to avoid collisions with obstacles. The experiment involved two conditions, with and without the aid of the ETA. Each condition was repeated for three trials, during which participants experienced a different VI (AMD, DR, and glaucoma) at a high level of severity.

We showed the effectiveness of the ETA in enhancing mobility performance of participants in terms of time to complete the task and number of collisions. Specifically, we tested two hypotheses: H1) the ETA can successfully assist subjects with simulated VI by reducing the time taken to complete the obstacle avoidance task; and H2) the ETA can successfully assist subjects with simulated VI by reducing the number of collisions occurred during the obstacle avoidance task. Within these hypotheses, we studied the percentage improvement in the time taken to complete the task (H1) and the difference in the number of collisions (H2) when using the ETA, for each participant and for each simulated VI.

We determined that the use of the ETA caused a significant reduction in completion time, in accordance with hypothesis H1. We also found that the VIs significantly impacted the completion time, suggesting the ability of a subject to complete the obstacle avoidance was affected by the specific VI we simulated. Precisely, we discovered that subjects performed trials faster with DR (mean value around 90 s) compared to AMD and glaucoma (mean values around 123 s and around 121 s, respectively). The difference in task completion time when experiencing DR compared to AMD and glaucoma can be explained by analyzing the symptoms of the three VIs. AMD primarily affects the central vision, which is extremely important for path planning. Glaucoma impairs peripheral vision, likely leading affected people to apply inappropriate gaze strategies during mobility tasks and therefore increasing the risk of collisions. These symptoms make it difficult for subject to gather comprehensive visual information in an environment, such that they are likely to bump into undetected obstacles. DR typically manifests with fluctuating vision, loss of detailed or sharp vision, blind spots, or loss of color vision. The greater residual vision of DR compared to AMD and glaucoma may have provided subjects with more confidence about the information gathered from the environment, thus decreasing completion time. However, we did not find a significant interaction between ETA and VI, indicating that the effectiveness of the ETA is consistent across VIs. Despite the different characteristics of each VI, the defects in the visual field functionalities, and the almost absence of residual vision, participants might feel safe traveling with route guidance provided by the devices alone, thus increasing the navigation speed. We propose that the device reduces the effort to concentrate on obstacle avoidance, thereby allowing participants to focus on better path planning.

We found that our ETA significantly reduced the total number of collisions in the obstacle avoidance task. However, we found no significant effect of the VI, when comparing the difference in the number of collisions with and without the ETA confirming consistency of the ETA across VIs. Similar to the previous metric, we attribute these findings to the symptoms of the simulated VIs. Not only does DR cause blurred, hazy, and distorted vision and seeing black lines and dots, but also it causes difficulties in perceiving depth, and negotiating steps and curbs (especially if easily distinguishable). These symptoms make it challenging to move in crowded places, avoid tripping, cross a street, and negotiate uneven or suspended objects. Likewise, glaucoma is well-known for its impact on mobility. In fact, the progressive restriction of the peripheral visual field in glaucoma strongly impairs clear identification of objects, which provides critical wide-field information about the environment. Additionally, symptoms such as blurriness, glare, and trouble differentiating boundaries and colors brought additional complexity during the obstacle avoidance task. As for AMD, due to the absence of residual central vision, it is even harder for subjects experiencing AMD to use eccentric viewing; thus, utilizing the ETA may not have been enough to negotiate the complex layout of the travel path. This behavior could have been partially influenced by the fact that participants used the device for the first time during this experiment. Preliminary training sessions about the proper use of the ETA could make participants more familiar with the device and more confident in their ability to properly interpret the information they received.

While our experiments provide a demonstration of our proof of concept of a VR platform for testing, refinement, and training with ETAs, our study is not free of limitations that should be addressed in more sophisticated and standardized versions of our platform.

- The performance assessment only focused on two metrics. Potential additional metrics that can be explored include eye gaze, head movements, and reaction time [44–47]. However, measuring these quantities would require more sophisticated acquisition systems.
- A limitation of utilizing a VR platform for the testing of ETAs is that we can only assess the performance of the feedback component of an ETA. The sensing component, in this case, is substituted by a sensing algorithm within the VR platform. Luckily, the testing of sensing systems is typically easier than that of feedback systems, whereby it does not require a human-in-the-loop.
- Another potential issue is that, while in a real environment subjects would be careful in avoiding obstacles to avoid hurting themselves, they do not have a similar instinct in VR. In VR a collision does not have any negative effect, so that subjects likely prioritized time to completion over the number of collisions. A potential way to address this issue is providing incentives, for example through rewards, for subjects to pay more attention to avoiding obstacles than finishing the experiment fast.
- The navigation modality implemented in the VR platform required users to explore the environment through the Oculus Touch controllers. This limitation does not allow to perfectly replicate the conditions faced in real-world implementations of our ETA, such as the confounding effects associated with the muscle contraction in the abdomen region during gait, which may negatively affect the discrimination of vibratory cues. We anticipate that the implementation of a controller-free navigation in a augmented reality (AR) setting will be part of extensions of our current study.

Overall, the main limitation of this study is represented by the lack of appropriate clinical data with which to constrain our simulated VIs. Despite the presence of studies that show simulation software ability to accurately replicate symptomps associated with VIs [45, 48, 49], the simulations described in the present work were only intended as an approximation of vision loss due to the three most widespread VIs. In future studies, the simulations could be improved by incorporating additional features of these VIs that are sometimes reported by patients, but which we currently lack the means to quantify robustly. Additionally, the simulations would also take into account the fact that, for many patients, the extent/quality of their vision loss may vary depending on their own physiological state or their current viewing conditions (such as ambient illumination). To objectively quantify the severity of simulated VIs, we propose a preliminary measure on the platform based on the standard definitions from the World Health Organization, which describes VIs as: i) mild when visual acuity is worse than 6/12 and equal to or better than 6/18 (i.e., seeing at 6 meters what the average person sees at 12 meters and so on); ii) moderate when visual acuity is worse than 6/18 and equal to or better than 6/60; and iii) and severe when visual acuity is worse than 6/60 and equal to or better than 3/60. These definitions, which are internationally accepted and incorporated into the International Statistical Classification of Diseases and Related Health Problems, are based upon testing of the vision with the Snellen chart. Potential corrections may be needed to account for the fact that the screen resolution does not currently match the eye’s foveal resolution. These corrections can be inferred from trials in VR without simulated VIs. Beyond VR, AR offers great opportunities for an intermediate and more realistic testing and training platform for ETAs. Such a step provides an even higher degree of immersion in the task, while still offering consistent stimuli and a safe and controlled environment. In our future work, we aim at integrating ETAs within AR systems.

To further evaluate our concept, we envision experiments designed to compare the two intervention methods (that is, VR-trained and traditionally trained), where both VI patients and orientation and mobility (O&M) specialists will be involved. In these experiments, we will also design a targeted VR and ETA training to be administered before performing the actual experiment. These experiments aim at testing the ability of the system to enhance navigation skills of persons with VI in a simulated complex urban scenario and aim at informing O&M trainers about the functioning and the proper use of an ETA. The inclusion of O&M specialists during the testing phases may provide useful insights to further improve the performance of ETAs, as well as promote a greater presence of training for best practise of these technologies.

In recent times, the development of VR applications in medical therapy, training, and rehabilitation has become increasingly important. Moreover, biomedical applications using VR technology are becoming increasingly accessible to consumers specialists as they proved to be both effective and intuitive. Our study represents a further step in this direction, toward the design and testing of ETAs for enhancing mobility of persons with VI. In addition to the testing capabilities, we believe that the use of immersive VR applications for training with ETAs functions as an engaging, motivating factor that can prolong the duration of exercises and hence the probability of effective therapeutic outcomes. The possibility to adapt the VR environment and tasks to the level and capacity of each patient or to deteriorate an underlying condition offers a further advantage for the success of the rehabilitation path or for preparing subjects for progressive loss of functions. The adaptability of the therapeutic approach is indeed critical in ensuring long-term engagement and progress.

## Methods

### Virtual reality software

We designed the VR platform in the Unity game engine, an ‘integrated development environment’ provided with interfaces that give access to all the tools needed for development in one place. The platform was designed through the Unity game engine for an Oculus Rift headset with Oculus Touch motion controllers. The users explored the environment through the controllers in a first-person perspective. The simulation of the three VIs described in Subsection “Interfacing VR and ETAs” was realized by combining the effects of two tools available in Unity, shader and culling mask. A shader is a small script that tries to approximate local light behavior on object surface. It can be used to modify levels of light, darkness, and color in a rendered image to achieve the desired outcome. A culling mask is a camera property used to generate photorealistic or non-photorealistic effects on parts of a scene separately. Symptoms of AMD were reproduced combining a Gaussian blur and distortion shaders. The typical gray spots that obscure the center of the visual field were added by means of a culling mask. Symptoms of DR were mimicked using a Gaussian blur shader and adding a culling mask to scatter dark spots throughout the visual field. Finally, glaucoma symptoms were achieved using a Gaussian blur shader as before but adding a culling mask that compromised the peripheral vision.

### Obstacles detection in VR

The design of the ETA consisted of a belt with ten discrete tactors to provide haptic feedback to the users’ abdomen. The tactors were realized using macro-fiber composites (MFCs), in the form of piezoceramic wafers with interdigitated electrodes on a polyimide film, and with structural epoxy layers. The MFC were bonded to a 54 × 20 × 0.25 (length × width × thickness) mm aluminum plate using epoxy to improve the performance of the actuators in terms of vibration amplitude and blocking force. The aluminum-backed MFC were then encapsulated in a 3D-printed case made of polylactic acid (PLA) to protect the actuator and prevent electric short circuits when a voltage on the order of kilovolts was applied across the electrodes. The tactors were mounted on a thin aluminum beam-based scaffold. The scaffold was in turn fastened to a commercial hiking belt that tethers in correspondence of the users’ hips. In particular, the actuators were disposed horizontally over six columns; the four central columns had two actuators each, separated by a vertical distance of 85 mm, while the two at the extremes of the belt had only one actuator, at in the middle of the belt vertically.

In our experiment, the ETA was connected to the PC through an Arduino Mega 2560 and interfaced with the VR platform by means of a C# script. This script used the serial port to allow a constant communication between an Arduino microcontroller and Unity. The Arduino used op-amps to drive the high-voltage amplifiers and control custom-printed astable multivibrator. The amplifiers controlled each of the ten actuators on the belt independently.

To make the obstacles detection possible in the virtual environment, we made use of a function called Raycast. Raycast is a physics function that projects rays into the scene, returning a Boolean value if a target is successfully hit. When a target is hit, from the one hand information about the correct actuators to activate was sent to the Arduino that, in turn, controlled high-voltage amplifiers that fed the actuators. From the other hand, information about the collision, such as the distance, position, or a reference of the object can be stored in a variable for further use. Considering our specific case, all rays were projected from the body of the player into the scene, such that the visual field was divided into a grid that resembled the arrangement of the actuators on the belt. These rays were split into groups: two rays were used for the detection of obstacles on the right and left of the player, and four groups of eight rays were used for the detection of obstacles located in the upper middle right, upper middle left, lower middle right, and lower middle left of the player, respectively. Each group of rays was linked to the corresponding actuator on the belt. The actuators vibrated when any obstacle was hit by at least one of the rays in the virtual environment. The length of the rays, which could be easily changed through the Raycast function, determined the range of action of the ETA. The frequency of vibration in the multivibrator circuit was regulated by a potentiometer based on the distance of the object from the user in VR.

### Metric quantification in VR

Two C# scripts were utilized to quantify and save the time to complete the task and total number of collisions in the virtual environment. The first script started and reset a stopwatch in each trial, while the second script simulated a collision by initiating the vibration of the Oculus Touch controller when the users were in contact with the virtual objects.

In the second script each virtual object was provided with a *RigidBody* and a *Collider* component. Specifically, we employed a *BoxCollider*, an invisible box-shaped primitive that encloses the object. When this box came in contact with the *Collider* of the player, it activated the vibration of the Oculus Touch Controller and registered a collision. For the player, we used a *CapsuleCollider* to allow the player to move in VR in a realistic manner.

### Participants

Forty-eight healthy participants recruited from the New York University (NYU) Tandon School of Engineering participated in the study. We followed strict exclusion criteria to avoid any discomfort due to the VR device. Specifically, participants excluded from this study included: people with significant cognitive dysfunction, previous neurological illness or psychiatric, heart condition or other serious medical condition, significant mobility restrictions, seizures, interfering medical devices, and pregnant or elderly individuals. All participants provided written informed consent in accordance with procedures approved by the Institutional Review Board (IRB) at New York University (NYU) Langone Health (IRB i21–00925).

### Statistical design

In the experiment, “with the ETA” and “without the ETA” were the two conditions tested for each type of VI. Apart from the specific VI and the use of the ETA, all trials were identically structured. Participants did not perform preliminary training on the VR platform, and they completed the experiment only once.

The order of conditions and trials were counterbalanced across participants. Specifically, there were three counterbalanced trials per condition. The counterbalancing had a two-fold purpose: i) to prevent the possibility that fatigue would mitigate the improvement brought about by the belt on users’ performance in the later stages of the experiment; and ii) to avoid biases due to prior knowledge about the platform or to familiarization with the device during the trials. Through this approach, we minimized learning and hindered time effects in the experiment.

### Statistical analyses

As a first step before performing statistical analyses, we conducted an outlier analysis on our datasets. Specifically, we used the box plot method on the raw data. The analysis revealed 21 outliers out of 288 trials in the completion time dataset and 17 outliers out of 288 trials for the number of collisions. For the purpose of keeping a balanced design for the statistical analyses, for each trial we decided to remove all data from these participants from the dataset.

We attribute the presence of outliers to cases where participants were inattentive to the vibration feedback of the ETA and increased the navigation speed. In fact, participants that resulted to be outliers had a very small completion time, but a very high number of collisions.

The statistical analyses were performed using RStudio version 2022.07.2. Specifically, the function *lmer* implemented in the package *lmerTest* version 3.1–3, and the function *anova* implemented in the package *rstatix* version 0.70 were used to perform the ANOVA including random effects. The functions *chisq.test* and *kruskal.test* implemented in the package *rstatix* version 0.70 were used to compute the *chi*-squared test and the Kruskal-Wallis test described in Subsection Experimental design and hypothesis testing respectively. The function *cor.test* implemented in the package *rstatix* version 0.70 was used to perform the Pearson’s correlation coefficients described in S1 Text. The graphical representation of the results showed in the violin plots in Subsection Mobility performance assessment was generated using the function *violinplot* implemented in MATLAB version R2022b while the scatter plots in S1 Text were created using the function *ggscatter* implemented in the package *ggplot2* version 3.3.6.

## Supporting information

S1 Text Analysis of the relationship between completion time and total number of collisions. (PDF)
