# Supplementary material for: Virtual reality as a means to explore assistive technologies for the visually impaired
Source: PLOS Digit Health. 2023 Jun 20;2(6):e0000275. doi: 10.1371/journal.pdig.0000275 (PMC10281573; doi:10.1371/journal.pdig.0000275)
Supplement: S1 Text — (PDF) [file pdig.0000275.s001.pdf]

Supporting information

S1 Analysis of completion time and total number of collisions

To understand whether subject preferred to optimize the time or to avoid the collision in order to complete the exercise, we evaluated the association between the time to complete the task and the number of collisions. We performed a correlation analysis by computing the Pearson's correlation coefficient, separately considering the two conditions tested, "with the ETA" and "without the ETA".

Such a correlation is likely due to participants' individual skills: those participants who experienced few collisions took less time to complete the task, while those who experienced many collisions required more time to complete the task.

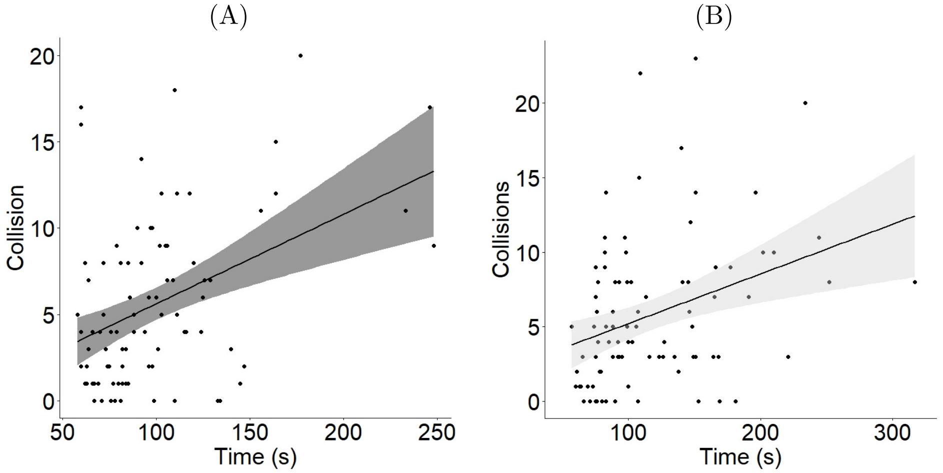

Fig. S1. Scatter plot of time taken to complete the task and number of collisions: (a) with the ETA and (b) without the ETA. Points correspond to individual realizations and the grey area represents the 95% confidence interval of the linear regression.
